# Outcome Measures in Evaluation of Weighted Blankets: A Scoping Review

**DOI:** 10.1155/oti/3663534

**Published:** 2025-02-19

**Authors:** Maria Lönn, Ellen Odéus

**Affiliations:** ^1^Department of Health and Care, School of Health and Welfare, Halmstad University, Halmstad, Sweden; ^2^Psychiatry Halland, Region Halland, Halmstad, Sweden; ^3^Department of Occupational and Physiotherapy, Queen Silvia's Children's Hospital, Sahlgrenska University Hospital, Region Västra Götaland, Gothenburg, Sweden; ^4^Institute of Neuroscience and Physiology, University of Gothenburg, Gothenburg, Sweden

**Keywords:** anxiety, investigative techniques, occupational therapy, research measurements, sleep disorders, weighted blankets

## Abstract

**Introduction:** Weighted blankets are an intervention used in healthcare settings for alleviating anxiety and sleep problems. Selection of appropriate outcome measures that capture relevant effects is important in clinical practice and research. However, outcomes used are diverse and not standardised. Therefore, this scoping review is aimed at identifying, synthesising, and describing available research and used outcome measures in research studies evaluating weighted blankets.

**Methods:** A scoping review was conducted, with a search of four databases. The search included studies published until February 2024. Results were categorized according to target population (adult or child) and primary outcome (sleep or anxiety). A conceptual map was developed to describe outcome measures used in the field.

**Results:** A total of 38 studies were identified, including 12 randomised controlled trials, 13 pre–post-studies, 6 case studies, 3 register studies, and 3 qualitative studies. The State–Trait Anxiety Inventory and the Anxiety Visual Analogue Scale were commonly used outcome measurements in evaluation of weighted blanket effects on anxiety and most commonly conducted in an adult population. There was considerable heterogeneity in the outcomes used, particularly within the domain of sleep. In studies evaluating weighted blankets as a sleep intervention for children, objective measurement methods such as actigraphy were common, as was the use of a sleep diary.

**Conclusion:** Even though there is extensive research available evaluating weighted blanket use and effects, there is a lack of standardised outcome measures and instruments are not adapted to use within occupational therapy practice. More research is necessary, informing clinical practice about which measures to use and when. Furthermore, there is a need to develop an instrument specifically tailored for use within occupational therapy practice to evaluate sleep interventions.

## 1. Introduction

The weighted blanket is a nonpharmacological sleep intervention used in occupational therapy settings to treat anxiety and sleep problems [1]. The weighted blanket is described to have a calming effect through deep pressure and sensory pathways [2]. It has also been noted to reduce restlessness, stress, and anxiety, thereby creating feelings of calm and safety [1, 3, 4]. However, outcomes reported in studies evaluating weighted blanket use are diverse and unstandardised.

The effect of weighted blankets can be understood through the interaction of arousal and environment [5]. Level of arousal, feelings of competence, and satisfaction in a specific environment are interconnected and indeed individual [6] and furthermore dependent on one's sensory system and ability to self-regulate [2, 7]. Sensory approaches are intended to improve different functional deficits such as attentional, emotional, motoric, communication, and social difficulties. Targeting an individual's sensory function is thus proposed to affect higher level domains, including motor symptoms [8], with potientially positive consequences on adaptive behaviour in a specific environment [5, 6]. The weighted blanket is thus used as a sensory approach to support self-management of distress [9], but it is also used as a sleep intervention to improve sleep [5, 10]. Individuals with sleep disturbances may benefit from using the weighted blanket as an aid for sleep onset, as part of a sleep routine, potentially targeting the individual's needs (such as difficulties with emotional and behavioural regulation) and thus improving sleep [11, 12].

A review published in 2020 [1] showed that weighted blankets may be effective in alleviating anxiety, which was also confirmed in a recent review published in 2024 [13]. However, both reviews concluded that evidence regarding the effect of weighted blankets on sleep are scarce, which partly is due to a heterogeneity in outcome measures used [1, 13]. New evidence has been published that supports the effects of weighted blankets on sleep, most recently by Lönn et al. [12]; Ekholm, Spulber, and Adler [14]; and Meth et al. [15]. Furthermore, a thesis on weighted blankets used as a sleep intervention among children with attention-deficit/hyperactivity disorder (ADHD) further demonstrated the use of weighted blanket, but the diversity of outcomes was also highlighted [5]. Although there is still a lack of a comprehensive review summarizing research concerning weighted blanket use according to design, population, and outcomes used, the lack of consistency in outcome measures used adds to the complexity of the field and furthermore impedes the possibility to conduct meta-analysis [1, 13]. Additionally, outcome measures differ when it comes to evaluation of weighted blankets according to age, which needs to be taken into consideration. Therefore, a scoping review of previously used outcome measures in evaluation of weighted blankets was warranted due to the complexity and heterogeneity of research in the field [16]. Knowledge gained from this review can thus be used for future research evaluating weighted blanket use, identify research gaps, and hopefully, improve understanding of which outcome measures to use that most appropriately captures the concept of interest.

The aim of this review was to identify, synthesise, and describe available research and the outcome measures used in studies evaluating weighted blankets. Additionally, an effort was made to categorise studies according to population (adult or child) and primary outcome (sleep or anxiety).

## 2. Material and Methods

This scoping review followed the guidelines in Preferred Reporting Items for Systematic reviews and Meta-Analyses (PRISMA) extension for scoping reviews (PRISMA-ScR) [17]. Tricco et al. [17] suggest that the review process adheres to five stages according to Arksey and O'Malley [18], which were followed in this review: identifying the research question; identifying the relevant studies; study selection; charting the data; and collating, summarizing, and reporting the results. A scoping review was chosen due to the diversity in design, variety of populations, and outcome measures used in research evaluating weighted blankets.

### 2.1. Identifying the Research Question

The research questions that guided the search were “What research is available within the scope of weighted blankets?” and “What outcome measures are used to assess anxiety and sleep in studies evaluating weighted blankets?”

### 2.2. Identifying the Relevant Studies

**Figure 1:**
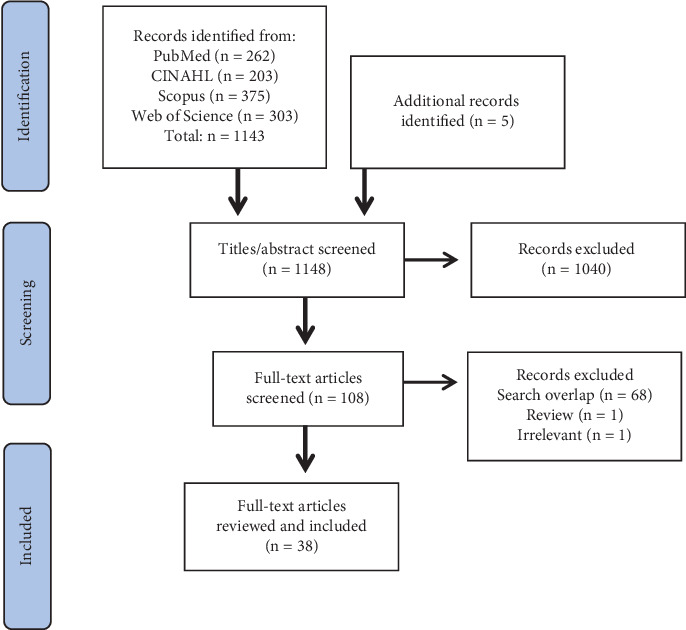
Flow chart of the selection process.

To identify relevant literature, the databases PubMed, CINAHL, Scopus, and Web of Science were systematically searched multiple times between January 2020 and February 2024, with a final search conducted in February 2024. References from identified articles were also reviewed to locate additional studies. Articles that met the inclusion criteria were catalogued in an Excel spreadsheet, detailing the authors, journal, year, title, and population. A final systematic search, conducted with assistance of a librarian, was performed in February 2024 across all databases using the following search terms: (blankets OR “deep pressure” OR “deep touch pressure”) AND (sleep OR anxiety OR arousal). The results of the final systematic search are presented in Figure 1 (identification).

### 2.3. Selecting the Studies

A review was conducted independently by the first two authors according to the inclusion criteria. Titles were screened (*n* = 1148), and abstracts were reviewed when necessary. Uncertain articles were flagged and discussed by the two authors and, if required, reviewed in full text before a final decision of exclusion was made. In total, 1040 articles were deemed irrelevant and excluded, and additional two articles were excluded for not meeting the inclusion criteria after full-text review. The remaining 38 articles, which were reviewed in full text, were deemed eligible and included in the scoping review.

The inclusion criteria were research evaluating the use or effectiveness of weighted blanket, English-language articles, studies involving adults or children aged > 1 year, and peer-reviewed research published between January 2000 and February 2024. Pilot studies were considered eligible if they met the inclusion criteria. The exclusion criteria were conference papers, reviews, and discussion/commentary papers. Both qualitative and quantitative studies were acceptable.

A consultation with an expert in the field was conducted to ensure that no relevant articles were overlooked in the search.

### 2.4. Charting the Data

Data were organised, and eligible criteria for the included studies were documented using an Excel grid. The summary of the data can be provided by the first author upon request. The 38 studies were examined in detail and data collected accordingly. From each of the included articles (*n* = 38), information was gathered on design, target group/age/setting, sample size, and used outcome measures related to anxiety or sleep.

### 2.5. Collating, Summarizing, and Reporting the Data

**Figure 2:**
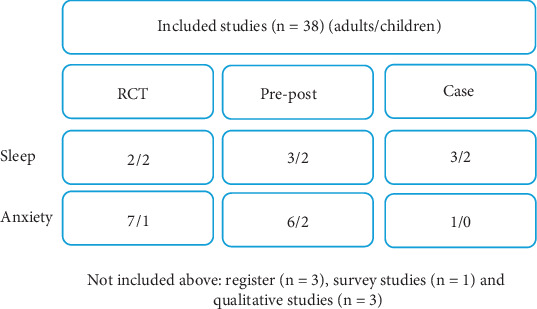
Breakdown of included studies.

Data were grouped according to population (child or adult), study type (qualitative study, register study, randomised controlled trial (RCT), and pre–post-study or case study), and primary outcome (anxiety or sleep). This information was summarized and presented in Figure 2. Outcome measurements used in the studies were also summarized and categorized by primary outcome (anxiety or sleep) and population (child or adult).

### 2.6. Conceptual Model Development

A conceptual model was developed based on the outcomes extracted from the scoping review. The model was developed in a deductive manner using a directed content analysis [19] and guided by the conceptual outcome model by Wilson and Cleary [20]. Common concepts and considerations of similarities and differences were grouped into subcategories and categories and illustrated in the conceptual model. Concepts were only included in the model if extracted to this scoping review, and the conceptual model may thus lack in important concepts and aspects beyond the scope of this review.

### 2.7. Positionality Statement

The authors of this review are occupational therapists, and both have experience of prescribing weighted blankets to children and/or adults. Both have worked in outpatient settings where weighted blankets have been prescribed as an assistive technology for use in the home environment. As a result, the authors are familiar with the research field which could present bias through the dual roles of researchers and practitioners. The authors might interpret data through the lens of their professional experiences. However, the review was conducted according to the PRISMA guidelines [17] and the review process adhered to the five stages according to Arksey and O'Malley [18]. Objectivity was furthermore ensured as the authors had no prior collaboration, belong to different institutions, and work in different regions and thus present different experiences concerning prescription of weighted blankets. Additionally, the review was conducted in a systematic manner, without preconceived notions affecting the review process.

## 3. Results

A total of 1143 articles were identified in the systematic search, of which 38 articles were deemed relevant for inclusion in the review (Figure 1). This scoping review included 12 RCTs, 13 pre–post-studies, six case studies, and one survey study that evaluated the use of weighted blanket with sleep or anxiety as a primary outcome measure (Figure 2). Pre–post-studies were mainly conducted during short-term use, and longitudinal studies were not identified in the present review. Additionally, 3 register studies and 3 qualitative studies were identified, providing further insights into weighted blanket use. Specifically, three qualitative studies evaluated the following: nursing staff experiences with weighted blankets [21], parent experiences of children using weighted blankets [22], and children's experiences of using weighted blankets [11]. The three register studies reported prescription rates based on various background characteristics such as age, gender, and diagnosis and also examined changes in the prescription of sleep medications based on register data [10, 23, 24].

The results from this scoping review were grouped and presented according to the primary outcome (anxiety or sleep). Although not within the scope of this study, outcomes related to feasibility and acceptability were frequently reported as additional outcome measures and are therefore also described narrative. In order to further describe outcome measures used within weighted blanket research, a conceptual map is presented.

### 3.1. Evaluating Anxiety

Studies evaluating anxiety and/or autonomic response have been conducted in diverse settings, including psychiatric inpatient units, eating disorder inpatient setting, emergency departments, chemotherapy units, dental clinics, and postoperative settings (Tables 1 and 2). Seven RCTs evaluated anxiety/autonomic response or pain in adults, and one RCT assessed autonomic response in adolescents.

**Figure 3:**
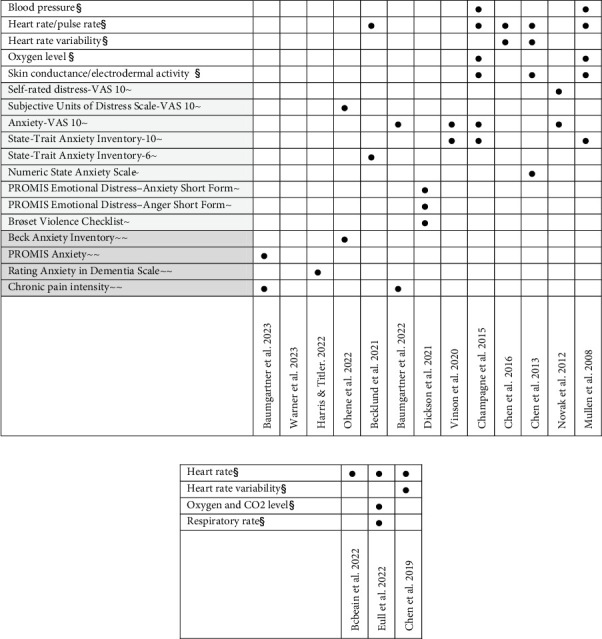
Outcome measures used in studies evaluating anxiety/autonomic response among (a) adults and (b) children. ^§^Biological and psychological variables and objectively measured outcomes; ^~^symptoms (proximal); ^~~^symptoms (distal).

Anxiety in adults was measured primarily through current anxiety assessments in experimental designs evaluating anxiety during weighted blanket use (described in the conceptual model as proximal measures). The most frequently used outcome measures were the State–Trait Anxiety Inventory (STAI; State) and the Anxiety Visual Analogue Scale (VAS) (Figure 3(a)). A few studies also evaluated more enduring changes (i.e., distal measures) in anxiety using the Beck Anxiety Inventory (BAI) (past month) and STAI (Trait). Current anxiety was the primary and most commonly used outcome measure in adults, typically assessed with the VAS. Various vital signs related to autonomic response were also frequently assessed in studies of weighted blanket use in experimental daytime settings, with heart rate being the most common measure. In children, assessment was limited to measures of autonomic response such as heart rate (Figure 3(b)). Subjective ratings of anxiety were not reported in studies evaluating weighted blanket use among children.

### 3.2. Evaluating Sleep

Similar to research on anxiety, studies evaluating the effects of weighted blankets on sleep have increased, with several new studies published in the last few of years (2020–2023). Our review identified a total of nine studies on adults (Table 3) and seven studies on children (Table 4), including one study that involved both adults and children [25].

Three qualitative studies were available, one evaluating children's experiences [11], one evaluating parents' experiences [22], and one evaluating nursing staff's experiences [21] (not included in the tables).

Research studies evaluating sleep in children included two crossover RCTs [12, 26], two noncontrolled open studies [27, 28], two single-subject designs [29, 30], a retrospective follow-up design [25], and two qualitative studies [11, 22]. In studies evaluating the effects of weighted blankets on children, with sleep as the primary outcome, only populations with autism and ADHD have been included (Table 4).

**Figure 4:**
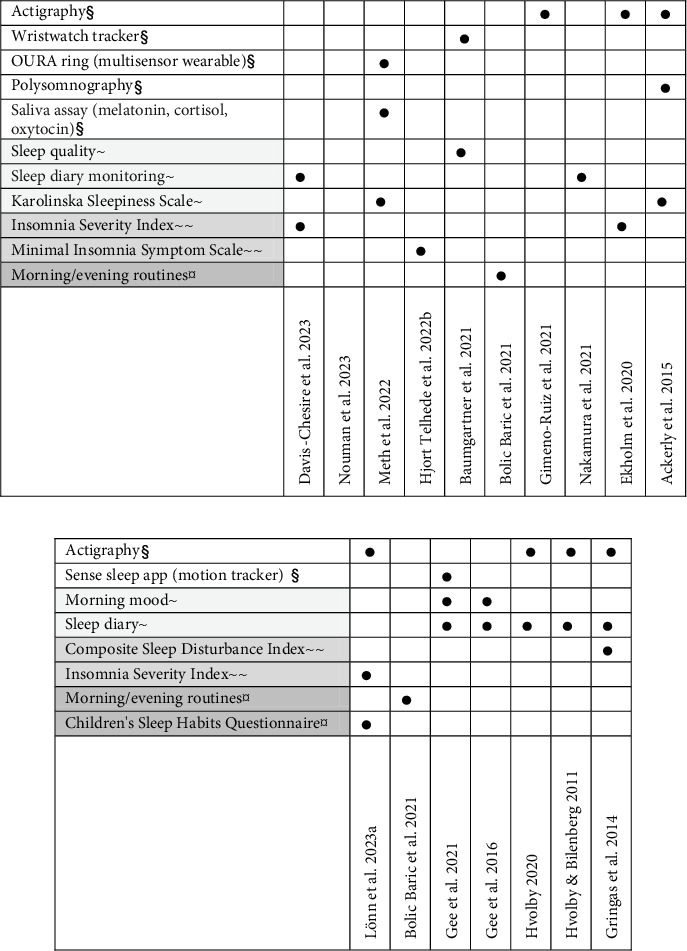
Outcome measures used in studies sleep among (a) adults and (b) children. ^§^Biological and psychological variables and objectively measured outcomes; ^~^symptoms (specific); ^~~^symptoms (global); ^¤^functional status.

A wide range of outcome measures have been used in studies evaluating the use of weighted blankets. The most commonly used method for measurement within sleep research among adults was actigraphy or some form of wearable motion/sensor tracker. Five studies employed an objective tracker (Figure 4(a)). The most frequently used validated questionnaires among adults were the Insomnia Severity Index (ISI) (two studies) and the Karolinska Sleepiness Scale (KSS) (two studies). The most commonly used method of measurement among children was actigraphy (four studies) and a sleep diary (five studies) (Figure 4(b)). In the evaluation of sleep using actigraphy, the most frequently reported outcomes were different measures of sleep patterns including total sleep time, sleep onset latency, wake after sleep onset, sleep efficiency and the number of awakenings.

### 3.3. A Conceptual Map of Anxiety and Sleep Outcomes in Weighted Blanket Research

**Figure 5:**
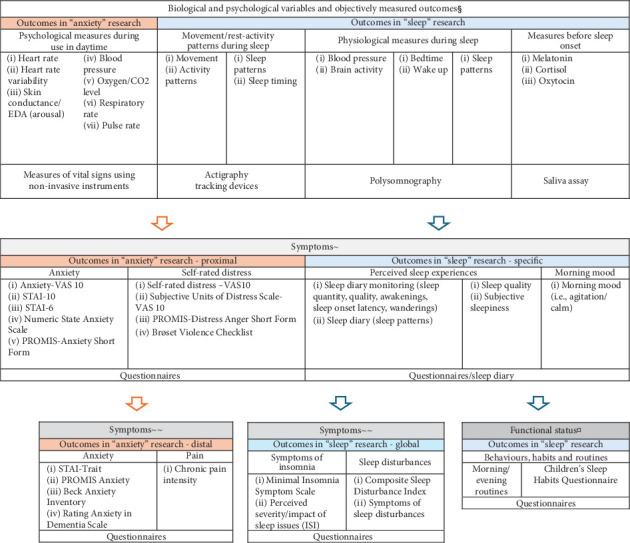
Conceptual map of anxiety and sleep outcomes used within weighted blanket research. Outcomes were categorized according to biological and physiological variables, symptoms, and functional status. ^§^Biological and psychological variables and objectively measured outcomes; ^~^symptoms (proximal or specific); ^~~^symptoms (distal or global); ^¤^functional status.

Outcome measures used in weighted blanket research were mapped to a conceptual model (Figure 5). Biological and psychological variables included variables where different objective methods are used such as actigraphy, tracking devices, polysomnography, saliva assay and measures of vital signs using non-invasive instruments. Symptoms within anxiety research were categorized according to proximal measures, used to evaluate anxiety and distress during actual use of the weighted blanket. Distal measures included outcomes reflecting a general perception during a longer time frame (such as anxiety symptoms during a week). Symptoms within sleep research were categorized according to specific and global symptoms. For example, specific measures such as sleep quantity and sleep timing reported in a sleep diary and global measures reflecting several aspects of sleep, such as the ISI. Sleep is a broader concept as compared to anxiety and thus included a larger variability of outcomes. Sleep outcomes that were categorized within functional status included outcomes reflecting behaviours, habits, and routines in connection to sleep and were limited to two studies in this scoping review [12, 25].

### 3.4. Evaluating Feasibility and Acceptability

Research evaluating weighted blanket use frequently reported outcomes related to feasibility and acceptability. For example, Ackerly, Badre, and Olausson [31] included specific questions about the blanket, such as satisfaction, settling, and security, while Mullen et al. [2] included questions about relaxation, weight, warmth, and the feel of the fabric. Additionally, other studies included questions regarding preference and comfort [3, 32]. Among children, questions about satisfaction [12] and behaviour during blanket use [26] were included alongside outcome measures related to sleep.

Nonadherence, side effects, and reasons for blanket removal were also noted in some studies [12, 33, 34].

## 4. Discussion

There has been an increase in research evaluating weighted blankets. This review identified 38 articles that fall within the scope of weighted blankets use, including RCTs, pre–post-studies, case studies, register studies, and qualitative studies. However, there is a considerable variability in the outcomes reported, as demonstrated in this scoping review, which encompasses both anxiety and sleep domains. This scoping review presents a conceptual model which describes outcome measures used within weighted blanket research. Outcome measures within the domain of anxiety have commonly been used within an adult population and included various aspects of distress, ranging from autonomic response to self-rated distress, anxiety, and pain. Outcome measures within the domain of sleep included both specific measures and more global measures and furthermore also measures related to individual behaviours, habits, and routines (i.e., functional status). It is clear from the conceptual map that more research in needed concerning potential sleep outcomes related to functional status. This is an important and relevant future research field for occupational therapists. The wide range of data collection methods, both objective and subjective, contributes to the complexity of using and recommending standardised instruments in both domains (sleep and anxiety). This review highlights the urgent need for standardised instruments and presents a first step forward mapping relevant outcomes when evaluating weighted blanket.

The results revealed a total of eight RCTs evaluating the effects of weighted blankets with anxiety or distress as an outcome measure. A meta-analysis has therefore been warranted to inform clinical practice in occupational therapy regarding the potential effects of weighted blankets. The review by Eron et al. [1] included three RCTs focused on anxiety and concluded that weighted blankets show promising effects. Furthermore, Wong et al. [13] recently demonstrated that weighted blankets are effective in reducing anxiety (standardised mean difference: −0.47) in a meta-analysis including five RCTs. However, Wong et al. [13] identified a limitation due to the heterogeneity in outcome reporting in the field, a concern also highlighted in the present review. The results from this review showed that physiological measures like heart rate/pulse rate were common. The most frequently used subjectively reported outcome measure to assess the effects of weighted blankets on anxiety were STAI (State) and anxiety VAS (both used in an adult population). Both of these measures assess anxiety during current use (i.e., proximal outcome measures). The anxiety VAS, in particular, is easy to administer and use, making it an appropriate outcome measure for evaluating weighted blanket use in both clinical practice and experimental settings [35].

The evaluation of long-lasting changes in anxiety levels (assessed through daily diaries or after a period of prolonged use, such as after 1 week or several weeks) is less common in weighted blanket research, according to the present review, and is thus warranted for future studies. In a published study protocol on ball blankets [36], the authors plan to use the BAI to assess long-term changes in anxiety, similar to the approach used by Ohene et al. [37]. For the evaluation of long-term anxiety changes in adolescents, BAI [38] or the STAI (Trait) [39] are two possible validated alternatives for assessing long-lasting anxiety changes in children and/or adolescents. This scoping review clearly presents a research gap concerning evaluation of anxiety or distress when using weighted blankets among children or adolescents.

Interestingly, although outside the scope of the review, outcome measures evaluating clients' sensory profiles were found to be uncommon. In fact, Scanlan and Novak [9] note a shift in the focus of sensory approach evaluations from perceptual-motor performance to outcomes related to distress and disturbed behaviour. Measurement of clients' sensory profiles was rare in the present scoping review, and when used, it was primarily for descriptive purposes or as an eligibility criterion [26, 29, 30]. Instruments serve various purposes, such as screening, diagnosis, evaluation, or measurement [40]. A client's sensory profile is not expected to change per se; however, their response to outcomes related to distress, anxiety, or disturbed behaviour may vary according to their sensory profile. A responder versus nonresponder analysis, incorporating measures of clients' sensory profiles, could be an intriguing area for future research. An important consideration in such analyses might be the type of weighted blanket used (e.g., fibre, chain, or ball blankets). Different clients may respond differently to weighted blankets based on sensory preferences [41], which may explain why certain types of blankets are preferred over others [10]. This represents an interesting avenue for future research. Furthermore, the conceptual map presented in this review could be extended to fully capture potential symptoms and impacts of weighted blankets, such as symptoms covering individuals' sensory profile, emotional status, cognition, and social abilities and additionally aspects covering daytime symptoms, function, and quality of life. This was however beyond the scope of this review.

The results from this scoping review showed that actigraphy, in combination with a sleep diary, was the most commonly used outcome measure in the evaluation of sleep among adults. In subjective evaluations with questionnairs, all six available studies used different questionnaires, some validated and others nonvalidated. The validated questionnaires included the ISI, KSS, and the Minimal Insomnia Symptom Scale. The diversity of instruments used impedes the possibility of conducting a meta-analysis, as they cover different aspects of sleep. Consensus is recommended, concerning relevant outcomes that fully capture adults and children's positive experiences of using weighted blankets. Qualitative research may be used in this process. For example, children described the potential of weighted blankets to induce calmness and safety when sleeping with a weighted blanket [11], though such measures have not, according to this review, been used in research evaluating sleep. The connection between anxiety and sleep research is absent, but nevertheless, this has been described as a possible link explaining the mechanism behind weighted blankets' effect on sleep [42] and thus an interesting future research field. There are overall heterogeneity in the evaluation of sleep interventions and a lack of standardised measures [43, 44]. The domain of sleep and rest has been largely neglected within occupational therapy research [45], and occupational therapists lack confidence in addressing sleep, despite acknowledging its importance for their clients [46, 47]. There is a need to develop instruments for use by occupational therapists in the domain of sleep and rest [47, 48]. Occupational therapists should use standardised instruments, as this is an essential part of conducting evidence-based practice [49]. Interestingly, only seven of the 38 available articles evaluating weighted blanket use as a sleep intervention were published in occupational therapy journals. Developing an instrument for use within occupational therapy practice to evaluate sleep interventions, such as weighted blankets, therefore seems warranted. The conceptual map developed for this scoping review also shows the urgent need for occupational therapists to contribute with knowledge to the field, for example, to further develop instruments that cover behaviours, habits, and routines related to the domain of sleep. This is a prerequisite for the further development of evidence-based occupational therapy practice in the domain of sleep.

This review identified objective measures, such as actigraphy, as the most commonly used method for evaluating sleep among children. However, there is a lack of evaluations of children's subjective sleep problems or sleep severity, with the exception of a crossover RCT, where a modified version of the self-reported ISI was used [12]. Previous research on parent-reported sleep problems includes the work of Gringras et al. [26], who used the Composite Sleep Disturbance Index (CSDI), and Lönn et al. [12], who used the Children's Sleep Habits Questionnaire (CSHQ). The CSHQ is the most frequently used outcome measure for parent-reported sleep problems in ADHD RCTs [42]. Some aspects of the CSHQ, such as items addressing various behaviours and habits, align well with the occupational therapy setting for evaluating children's sleep. However, the CSHQ is validated for use with school-aged children (4–10 years) [50] and may therefore be less suitable for older children and adolescents, due to differences in the presentation of sleep problems [51]. Other instruments for evaluating sleep among children and adolescents—particularly those that assess not only sleep-related emotions and behaviours but also habits and routines—represent a potential scope for future review. Such a review could provide valuable guidance to occupational therapists in addressing the domain of sleep. This review identified several studies reporting outcomes related to feasibility and acceptability. However, the measures used were not standardised and were rarely validated. Nevertheless, outcomes related to acceptability, such as satisfaction, preferences, and adherence, are crucial in intervention research [52]. This area aligns well with client-centred occupational therapy practice. Furthermore, measures of acceptability and adherence are commonly used in the evaluation of assistive technology [53]. In fact, satisfaction, functional performance, and usage are the most frequently used outcome measures in occupational therapy research [53], and their relevance has been shown in the evaluation of weighted blanket use as a sleep intervention [5, 12, 54]. The inclusion of validated questionnaires covering aspects such as acceptability and adherence could, therefore, be an important complement to the evaluation of the effects of weighted blankets [26, 54].

### 4.1. Limitations

This review did not document primary articles on the instruments used, and no bias assessment or psychometric evaluation was conducted. This represents a limitation and an area for further research. However, interested readers can find relevant documentation within the studies included in this scoping review. The 38 included studies are available in a supporting table. Outcomes related to daytime function or sensory profiles were not collected, which is another limitation. Additionally, measures related to acceptability were not systematically presented, as this was outside the scope of the current study. However, we recommend further research in this area.

The studies included in this review span diverse settings and populations, which may also be a limitation. Not all outcome measures may be appropriate for every context. Moreover, other validated instruments not covered in the studies identified in this review may exist. This review presents potential outcome measures for evaluating the effects of weighted blankets on anxiety or sleep outcomes. We propose that future research should consider qualitative studies, as these may provide additional insights into potential targets for evaluating the effects of weighted blankets.

## 5. Implications for Occupational Therapy

• STAI (State) and anxiety VAS may be appropriate outcome measures for evaluating the proximal effects of weighted blanket on anxiety.
• For the evaluation of long-lasting effects on anxiety, such as in outpatient settings, outcome measures like the BAI and STAI (Trait) are suggested as possible outcome measures.
• Evaluation of weighted blankets' effects on anxiety/distress among children and or adolescents is an unexplored area, and more research is thus needed.
• More research is needed to identify appropriate instruments for evaluating sleep, particular those that are suitable for occupational therapy practice. Such instrument should address not only sleep-related emotions and behaviours but also habits and routines associated with sleep.

## 6. Conclusion

This review provides clinicians with synthesised information on potential outcomes for evaluating the effects and use of weighted blankets. It extends previous reviews by offering a comprehensive mapping of weighted blanket use in relation to both anxiety and sleep outcomes. While the results demonstrate the volume of available research, the heterogeneity of outcomes used is a notable limitation. This review can inform the research community and help standardise outcome measures for evaluating weighted blanket use. Additionally, it highlights the need for outcome measures related to sleep that are relevant to occupational therapy practice and research.

## Figures and Tables

**Table 1:** Articles with anxiety/autonomic response as primary outcome in adults (*n* = 14).

**Authors**	**Year**	**Population/setting**	**Design**	**N**
Baumgartner et al.	2022^a^/2023	Chronic pain sample	RCT	47 + 48
Warner et al.	2023	Trauma patients	Pre–post	12 + 12
Harris and Titler	2022	People with dementia and caregivers	Pre–post	21
Ohene et al.	2022	Eating disorder inpatient setting	RCT	11 + 12
Becklund et al.	2021	Inpatient mental health hospital	Pre–post	61 + 61
Dickson et al.	2021	Emergency department	Pre–post	15
Vinson et al.	2020	Chemotherapy infusion session	Crossover RCT	58
Losinski et al.	2017	Students with autism	Case	3
Champagne et al.	2015	Inpatient psychiatric setting	Crossover RCT	30
Chen et al.	2016	Dental setting	Crossover RCT	60
Chen et al.	2013	Dental setting	Pre–post	15
Novak et al.	2012	Inpatient psychiatric clinic, sensory room	Pre–post	75
Mullen et al.	2008	Experimental setting	Crossover RCT	32

Abbreviations: ADHD, attention deficit hyperactivity disorder; RCT, randomised controlled trial.

^a^Baumgartner et al. [3] also included sleep outcome measures; however, pain/anxiety was the primary outcome.

**Table 2:** Articles with anxiety/autonomic response or feasibility as primary outcome in children (*n* = 3).

**Authors**	**Year**	**Population/setting**	**Design**	**N**	**Age**
Bcbeain et al.	2022	Children, stabilisation sedation dental visits	Pre–post–chart review	24	Mean age 6.5
Eull, Zachrison, and Nickel	2022	Children, postoperative setting	Pre–post	93	Mean age 5.2
Chen et al.	2019	Adolescents, dental setting	RCT	38	Mean age 15.6

Abbreviation: RCT, randomized controlled trial.

**Table 3:** Articles with sleep as primary outcome measure in adults (*n* = 9).

**Authors**	**Year**	**Population/setting**	**Design**	**N**
Davis-Chesire et al.	2023	Adults with insomnia severity and sensory sensitivity	Case	5
Nouman et al.	2023	Adults with periodic leg movement syndrome	Case	1
Meth et al.	2022	Healthy adults	Crossover RCT	26
Hjort Telhede et al.	2022b	Older individuals in nursing homes	Pre–post	68
Bolic Baric et al.	2021	Children and adults with ADHD/autism	Survey	85
Gimeno-Ruiz et al.	2021	Adults with intellectual disability	Pre–post	64
Nakamura et al.	2021	Older individual with Alzheimer disease	Case	1
Ekholm, Spulber, and Adler	2020	Adults with insomnia and psychiatric conditions	RCT	56+64
Ackerley et al.	2015	Adults with insomnia	Pre–post	31

Abbreviations: ADHD, attention deficit hyperactivity disorder; RCT, randomised controlled trial.

**Table 4:** Articles with sleep as primary outcome measure in children (*n* = 7).

**Authors**	**Year**	**Population/setting**	**Design**	**N**	**Age**
Lönn et al.	2023a	Children with ADHD	Crossover RCT	94	6–14
Bolic Baric et al.	2021	Children and adults with ADHD/autism	Survey	85	< 17
Gee et al.	2016/2021	Children with autism	Case	2/2	4–5
Hvolby	2020	Children with ADHD	Pre–post	36	8–13
Gringras et al.	2014	Children with autism	Crossover RCT	32 + 35	5–16
Hvolby and Bilenberg	2011	Children with ADHD	Pre–post	21 + 21	8–13

*Note:* Lönn et al. 2023a was published online in 2023 and later assigned an issue in 2024. Lönn et al. are referred to in the reference list as Lönn et al. 2024 [12] and in the supporting table as Lönn et al. 2023a.

Abbreviations: ADHD, attention deficit hyperactivity disorder; RCT, randomised controlled trial.

## Data Availability

The data that support the findings of this study are available from the corresponding author upon reasonable request.
