# Supplementary material for: Outcome Measures in Evaluation of Weighted Blankets: A Scoping Review
Source: Occup Ther Int. 2025 Feb 19;2025:3663534. doi: 10.1155/oti/3663534 (PMC11865465; doi:10.1155/oti/3663534)
Supplement: Supporting Information — Additional supporting information can be found online in the Supporting Information section. Articles included in review. [file 3663534.f1.pdf]

Supplementary 1. Articles reviewed and included in the scoping review of weighted blankets.

Authors	Year	Titel	Journal
Mullen et al.	2008	Exploring the Safety and Therapeutic Effects of Deep Pressure Stimulation Using a Weighted Blanket	*Occupational Therapy in Mental Health*
Hvolby & Bilenberg	2011	Use of Ball Blanket in attention-deficit/hyperactivity disorder sleeping problems	*Nord J Psychiatry*
Novak et al.	2012	Pilot study of a sensory room in an acute inpatient psychiatric unit	*Australas Psychiatry*
Chen et al.	2013	Physiological Effects of Deep Touch Pressure on Anxiety Alleviation: The Weighted Blanket Approach	*Journal of Medical and Biological Engineering*
Gringras et al.	2014	Weighted blankets and sleep in autistic children--a randomized controlled trial.	*Pediatrics*
Ackerley et al	2015	Positive Effects of a Weighted Blanket on Insomnia	*Journal of Sleep Medicine & Disorders†*
Champagne et al.	2015	Evaluating the Safety and Effectiveness of the Weighted Blanket With Adults During an Inpatient Mental Health Hospitalization	*Occupational Therapy in Mental Health*
Chen et al.	2016	Effect of deep pressure input on parasympathetic system in patients with wisdom tooth surgery	*Journal of the Formosan Medical Association*
Gee et al.	2016	Improving sleep quality using weighted blankets among young children with an autism spectrum disorder	*International Journal of Therapy and Rehabilitation*
Losinski et al.	2017	The Effects of Deep Pressure Therapies and Antecedent Exercise on Stereotypical Behaviors of Students With Autism Spectrum Disorders	*Behavioral Disorders*
Chen et al.	2019	Parasympathetic effect of deep pressure input on third molar extraction in adolescents.	*J Formos Med Assoc*
Ekholm et al	2020	A randomized controlled study of weighted chain blankets for insomnia in psychiatric disorders	*J Clin Sleep Med*
Hvolby, A.	2020	The Application of Ball Blankets in the Treatment of Sleeping Difficulties in Children with Attention Deficit/Hyperactivity Disorder. Effect on Quality of Life and Daily Functioning.	*J Sleep Med Disord†*
Vinson et al.	2020	Weighted Blankets: Anxiety Reduction in Adult Patients Receiving Chemotherapy.	*Clin J Oncol Nurs*
Becklund et al.	2021	Using weighted blankets in an inpatient mental health hospital to decrease anxiety	*Journal of Integrative Medicine*
Bolic Baric et al.	2021	The effectiveness of weighted blankets on sleep and everyday activities - A retrospective follow-up study of children and adults with attention deficit hyperactivity disorder and/or autism spectrum disorder	*Scand J Occup Therapy*
Dickson et al.	2021	Effectiveness of the Weighted Blanket With Psychiatric Patients in the Emergency Department: A Pilot Study.	*J Am Psychiatr Nurses Assoc*
Gee et al.	2021	Weighted Blankets and Sleep Quality in Children with Autism Spectrum Disorders: A Single-Subject Design	*Children (Basel)*
Gimeno-Ruiz et al.	2021	Effects of Deep Propioceptive Stimulation on the Efficiency of Sleep in People with Intellectual Disability and the Necessity of Extensive-Generalized Support	*Journal of Sleep Medicine & Disorders†*
Nakamura et al.	2021	A case of effective usage of a weighted blanket for a person with severe dementia	*Psychogeriatrics*
Baumgartner et al.	2022	Widespread Pressure Delivered by a Weighted Blanket Reduces Chronic Pain: A Randomized Controlled Trial	*J Pain*
Bcbeain et al.	2022	Weighted Blanket Use as an Alternative to Protective Stabilization During Moderate Sedation	*PEDIATRIC DENTISTRY†*
Eull et al.	2022	Feasibility trial of weighted blankets as an intervention for emergence delirium in postoperative pediatric patients	*J Pediatr Nurs*
Harris & Titler	2022	Feasibility and Acceptability of a Remotely Delivered Weighted Blanket Intervention for People Living With Dementia and Their Family Caregivers	*J Appl Geront*
Meth et al.	2022	A weighted blanket increases pre-sleep salivary concentrations of melatonin in young, healthy adults	*J Sleep research*
Ohene et al.	2022	Assessing the Impact of Weighted Blankets on Anxiety for Patients With Eating Disorders in an Inpatient Setting: A Randomized Control Trial Pilot	*The American Journal of Occupational Therapy*
Baumgartner et al.	2023	Chronic pain patients low in social connectedness report higher pain and need deeper pressure for pain relief	*Emotion*
Davis-Chesire et al.	2023	The impact of WB use on adults with sensory sensitivity and insomnia	*Occupational Therapy International*

Lönn et al.	2023a	The efficacy of weighted blankets for sleep in children with attention-deficit/hyperactivity disorder-A randomized controlled crossover trial	*J Sleep research*
Lönn et al.	2023b	Experiences of Using Weighted Blankets among Children with ADHD and Sleeping Difficulties	*Occupational Therapy International*
Nouman et al.	2023	Weighted Blanket Therapy for Periodic Limb Movement Disorder: A Case Report Highlighting Improved Sleep Quality and Reduced Symptoms	*Cureus*
Warner et al.	2023	Weighted Blankets for Pain and Anxiety Relief in Acutely Injured Trauma Patients	*J pain palliat care pharmacotherapy*
Hjort Telhede et al.	2022a	Nursing staff's experiences of how weighted blankets influence resident's in nursing homes expressions of health	*Int J Qual Stud Health Well-being*
Hjort Telhede et al.	2022b	Weighted Blankets' Effect on the Health of Older People Living in Nursing Homes	*Geriatrics (Basel)*
Cederlund et al.	2023	The introduction of a weighted blanket was not associated with a reduced collection of pharmaceuticals prescribed for sleep disturbances in children: A register-based study	*Sleep Med*
Odéus et al.	2022	Weighted blankets for sleep problems - prescription, use and cost analysis	*Scand J Occup Therapy*
Steingrimsson et al.	2021	Weighted blanket and sleep medication use among adults with psychiatric diagnosis - a population-based register study	*Nord J Psychiatry*
Larsson et al.	2023	Parents' Experiences of Weighted Blankets' Impact on Children with Attention-Deficit/Hyperactivity Disorder (ADHD) and Sleep Problems-A Qualitative Study	*Int J Environ Res Public Health*

†Scientific level 0/or not found in the Norwegian list. https://kanalregister.hkdir.no/publiseringskanaler/Forside.action?request_locale=en
